# Analysis of the Mouse Y Chromosome by Single-Molecule Sequencing With Y Chromosome Enrichment

**DOI:** 10.3389/fgene.2020.00406

**Published:** 2020-05-07

**Authors:** Yuki Yano, Tomoki Chiba, Hiroshi Asahara

**Affiliations:** ^1^Department of Systems BioMedicine, Tokyo Medical and Dental University, Tokyo, Japan; ^2^Department of Molecular and Experimental Medicine, The Scripps Research Institute, San Diego, CA, United States

**Keywords:** mouse Y chromosome, fluorescence-activated cell sorter (FACS), long-read sequencing, MinION, gap closing

## Abstract

Since human and mouse Y chromosomes contain repeated sequences, it is difficult to determine the precise sequences and analyze the function of individual Y chromosome genes. Therefore, the causes of many diseases and abnormalities related to Y chromosome genes, such as male infertility, remain unclear. In this study, to elucidate the mouse Y chromosome, we enriched the mouse Y chromosome using a fluorescence-activated cell sorter (FACS) equipped with commonly used UV and blue 488 nm lasers and read the nucleotides using the Oxford Nanopore MinION long-read sequencer. This sequencing strategy allows us to cover the whole known region as well as the potential undetermined region of the Y chromosome. FACS-based chromosome enrichment and long-read sequencing are suitable for analysis of the Y chromosome sequences and may lead to further understanding of the physiological role of Y chromosome genes.

## Introduction

Male mammals have a Y chromosome, but its length and the encoded genes vary greatly among species (Hughes and Page, 2015). Since the Y chromosome does not have a homologous chromosome, recombination with the X chromosome occurs only in the region near the telomere, due to partial sequence similarity observed locally (Raudsepp and Chowdhary, 2015). Therefore, it has been considered that the Y chromosome is much more susceptible to genomic deletions and mutations than other chromosomes (Haldane, 1947). Although the human genome project was completed 15 years ago (Consortium, 2004), the complete sequence of the Y chromosome has not been deciphered for several reasons, including the high degree of repeated sequences and the presence of large regions of heterochromatin. Mice are often used as a model organism, and a draft sequence of the mouse genome was published without enough Y chromosome data in 2002 (Waterston et al., 2002). Thereafter, the mouse Y chromosome was re-sequenced and reported in 2014 using a BAC library covering the mouse Y chromosome (Soh et al., 2014). However, the complete genomic sequence of the mouse Y chromosome remains unclear because of the ampliconic region characterized by repeated sequences, which covers 98% of the chromosome (Soh et al., 2014).

Functional analysis of individual genes of the Y chromosome has also remained a challenge. Due to the extensive presence of repeated sequences, it has been difficult to utilize the conventional gene-targeting method of homologous recombination in embryonic stem cells. Recent applications of gene-editing technologies, such as TALEN and CPISPR/Cas9, enabled us to examine the precise function of each gene in the Y chromosome. In this regard, we have successfully produced and analyzed a series of knockout (KO) mice for Y chromosome-related genes and revealed critical functions of the genes *in vivo*, including the role of Sry in sex differentiation and eIF2s3y, Zfy1 and 2 in spermatogonia (Kato et al., 2013; Wang et al., 2013a, b; Matsubara et al., 2015; Nakasuji et al., 2017). In addition to single or duplicated genes, the Y chromosome also contains multi-copy genes of undetermined copy number, such as Rbmy. To further understand the physiological function of the Y chromosome, it is important to elucidate the precise copy number and location of multi-copy genes, which will allow us to generate KO mice precisely modified for each gene’s copy number.

Third-generation sequencers, including Pacific Biosciences and Oxford Nanopore Technology MinION, have emerged (van Dijk et al., 2018) that harbor technologies able to process extensive repeat sequences. These sequencers enable direct sequencing of a single molecule of DNA and/or RNA in real time without PCR amplification. More importantly, MinION sequencing has been reported to produce read length up to 882 kb (Jain et al., 2018). Given that the input molecule can be prepared without DNA synthesis or PCR amplification, these sequencers can detect nucleotide modifications, such as cytosine and adenosine methylation, and prevent biases derived from repeated sequences (Rhoads and Au, 2015; Kono and Arakawa, 2019). Recent studies have attempted to uncover the genomic region, such as by looking at highly repeated sequences and structural variants (Jain et al., 2018; De Coster et al., 2019). However, there remain many genes with unknown functions and regions where the nucleotide sequence is not elucidated on the Y chromosome.

In this study, we attempted to analyze the sequences of the mouse Y chromosome using Fluorescence-Activated Cell Sorting (FACS)-based chromosome enrichment in combination with the Oxford Nanopore MinION sequencer. RAW264.7 cells, which are derived from the BALB/c male mice widely used for biomedical research, were used as a source. Here, we report that Y chromosome enrichment was performed using UV and blue 488 nm lasers and that long-read nucleotide sequences could be obtained from sorted chromosomes by the MinION sequencer. In addition, we generated contigs and analyzed undetermined sequences of the mouse Y chromosome. These results could demonstrate a way to decipher the undetermined chromosome sequence by using a cell sorter equipped with commonly used UV and blue 488 nm lasers in combination with a long-read sequencer.

## Materials and Methods

### Cell Culture

RAW264.7 cells were cultured in Dulbecco’s modified Eagle’s medium (Sigma-Aldrich) supplemented with 10% fetal bovine serum (Gibco) at 37°C in 5% CO_2_.

After 24 h incubation, RAW264.7 cells were treated with Colcemid (0.05 μg/mL, Nakarai Tesque) for 12 h.

### Preparation of M Phase Chromosomes

M phase cells were brought to suspension by tapping the bottom of the dish. The supernatant was collected in 50 mL Falcon tubes and centrifuged for 3 min at 1,200 rpm at room temperature. The pellet was washed with 1 × PBS and centrifuged for 3 min at 1,200 rpm at room temperature. The washed pellet was resuspended by pipetting in hypotonic solution (50 mM KCl, 10 mM MgSO_4_, 5 mM HEPES, 0.25 mg/mL RNase A, 3 mM Dithiothreitol) and incubated at room temperature for 10 min. Then, Triton X-100 was added to the suspension for a final concentration of 0.25% and incubated for 10 min at room temperature. Aliquots of 1 mL of the suspension were transferred to 1.5 mL DNA LoBind tubes (Eppendorf) and passed 10 times through a 27G needle using a 1 mL syringe. The suspension was added to hypotonic propidium iodide (Waterston et al., 2002) and Hoechst33342 solution (50 μg/mL PI, 0.1 μg/mL Hoechst33342, 50 mM KCl, 0.1% sodium citrate, 0.2% NP–40, 0.25% RNase A) and stained for 30 min on ice in the dark.

### Sorting and Collection of Y Chromosome

**FIGURE 1:**
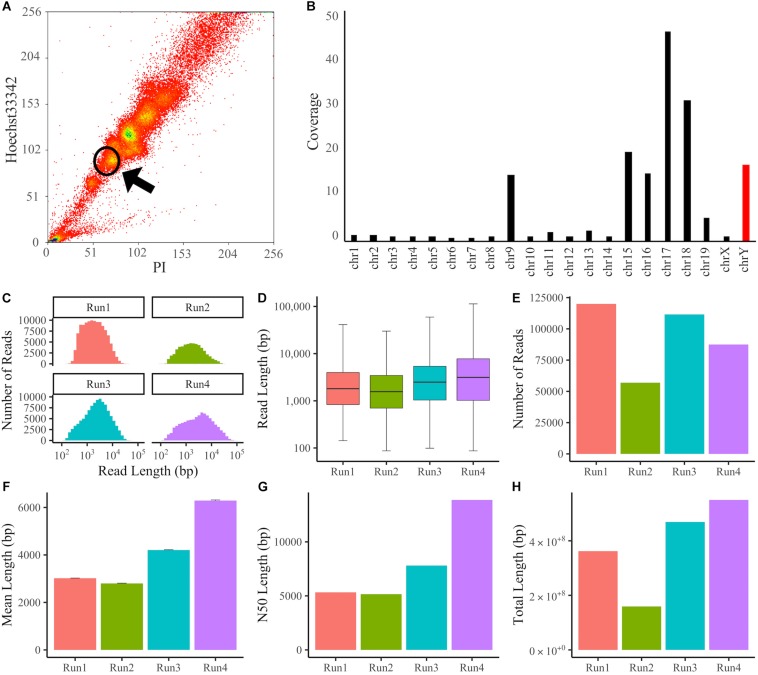
Sorting of chromosomes and Y chromosome data following MinION sequencing. **(A)** Flow Karyotyping of the RAW264.7 genome. Chromosomes were dyed with PI and Hoechst33342. Y chromosomes were collected from the circled area. **(B)** Coverage of chromosomes mapped using BWA-MEM. The Y chromosome was enriched with 16.8x coverage. **(C–H)** The Y chromosome sequencing data from individual experiments. **(C)** Histogram of read length. **(D)** Boxplot of read length. **(E)** Number of reads. **(F)** Mean length. Error bars denote standard error (SE). **(G)** N50 length. **(H)** Total length.

The dyed suspension was passed through a 35 μm nylon mesh and sorted using a MoFlo XDP FACS (Beckman Coulter). Karyotyping was performed using a 100-μm nozzle and two standard lasers: an air-cooled blue 488 nm solid-state laser and an air-cooled UV 355 nm solid-state laser. PI fluorescence was collected with a 625/26 filter, and Hoechst33342 fluorescence was collected with a 450/65 filter. Data were acquired at a rate of 10,000–15,000 events/s and analyzed with Summit software v5.3.0. Dot plotting was performed with two parameters, PI vs. Hoechst, and the region surrounded by a circle was collected (Figure 1A).

### DNA Purification and Library Preparation

The sorted DNA solution was added to an equal volume of isopropyl alcohol and centrifuged at 9,500 rpm for 30 min. The pellet of sorted DNA was resuspended using 1 × PBS and purified using a MagAttract HMW DNA Kit (QIAGEN). The DNA concentration was measured using a Qubit 2.0 fluorometer (Thermo Fisher Scientific) and adjusted to 1 μg/45 μL. The library was prepared from purified DNA according to the Ligation Sequencing Kit (SQK-LSK108) protocol (Oxford Nanopore Technologies). DNA (1 μg/45 μL) was mixed with 7 μL NEBNext Ultra II End Prep Reaction Buffer (NEB), 3 μL NEBNext Ultra II End Prep Enzyme Mix (NEB), and 5 μL nuclease-free water. The sample was incubated for 5 min at 20°C and for 5 min at 65°C. The incubated sample was purified using 60 μL AMPure XP beads (Beckman Coulter) and eluted in 31 μL nuclease-free water. The concentration of 1 μL of eluted DNA was measured using the Qubit 2.0 fluorometer (Thermo Fisher Scientific). The 30 μL end-prepped DNA solution was mixed with 20 μL Adapter Mix 1D and 20 μL Blunt/TA Ligase Master Mix. The sample was incubated for 30 min at room temperature. The incubated sample was purified using 60 μL AMPure XP beads and washed using 140 μL ABB Buffer (Oxford Nanopore Technologies). The purified DNA was eluted after adding 15 μL nuclease-free water for 10 min. One microliter of eluted DNA was used to measure its concentration using the Qubit 2.0 fluorometer (Thermo Fisher Scientific). The priming buffer was prepared by mixing 12 μL DNA library, 35 μL Running Buffer with Fuel Mix (Oxford Nanopore Technologies), 25.5 μL Library Loading Bead Kit (Oxford Nanopore Technologies), and 2.5 μL nuclease-free water. The mixture was loaded to the flowcell with MinKNOW v1.11.5–v3.3.2.

### Basecalling and Mapping

The FAST5 files of MinION raw sequencing data were basecalled using Guppy v3.1.5. The commands used in this study were described in Supplementary Data Sheet S1. The basecalled data in FASTQ files were mapped to the GRCm38.p6 genome using BWA-MEM v0.7.1 with the -x ont2d option (Li, 2013). This option is optimized for mapping the nanopore reads. Mapping data in the SAM files were sorted in the BAM format and indexed using SAMtools v1.9 (Li et al., 2009). The error rate was calculated by comparison of the GRCm38.p6 Y chromosome sequence and the mapped BAM files using AlignQC v2.0.5 (Weirather et al., 2017).

### *De novo* Assembly

The basecalled data in FASTQ files were assembled using Flye v2.4.2 (Kolmogorov et al., 2019). The genome size was assumed to be 92 Mb for the mouse Y chromosome. The raw contigs in the assembled data were polished using minimap2 v2.17 with the -x map-ont option (Li, 2018) and Racon v1.4.3 with default parameters (Vaser et al., 2017). This minimap2 option is optimized for finding overlap between the nanopore reads. This process was repeated three times. Minimap2 could generate a PAF (Pairwise mApping Format) file from a target file and a query file. Racon required a contig file, a reads file, and a PAF file. The polished contigs were mapped to the reference genome or the newly created genome by LR_Gapcloser using BWA-MEM with the -x ont2d option. Mapping data in SAM files were sorted using the BAM format and indexed using SAM tools. Contigs mapped to each chromosome in FASTA files were extracted from the mapped BAM files and compared with the Y chromosome sequence of the GRCm38.p6 genome or the newly created genome by LR_Gapcloser using D-GENIES online (Cabanettes and Klopp, 2018). D-GENIES requires the target sequence and query sequence in a FASTA file.

### Gap Closing of the Y Chromosome

The gap sequence of the Y chromosome of GRCm38.p6 was closed using LR_Gapcloser with -s n -r 8 options (Xu et al., 2019). LR_Gapcloser required a reference sequence containing gaps in a FASTA file and reads in a FASTA/FASTQ file. The input reads were fragmented into short tags of equal length and aligned using the BWA-MEM algorithm. LR_Gapcloser selected the reads that span a gap and filled the gap sequence of the reference genome with the selected sequences of the input reads. Then, the raw reads and the contigs assembled by Flye were re-mapped to the new gap-closed genome using BWA-MEM with the -x ont2d option. Focusing on the 10–20 kb before and after the gap-closed region, the BAM files mapped to the reference genome and the gap-closed genome were manually compared using the Integrative Genomics Viewer (Robinson et al., 2011).

## Results

### Y Chromosome Enrichment and MinION Sequencing

To enrich the Y chromosome, monocyte/macrophage RAW264.7 cells derived from BALB/c mice were treated with Colcemid to arrest the cell cycle at the M phase, and Y chromosomes were sorted by FACS according to Hoechst33342 and PI fluorescence intensities (Figure 1A). Then, DNA was purified with silica-coated magnetic beads to prevent DNA fragmentation. We sequenced the DNA using Oxford Nanopore MinION flowcells and obtained sequencing data of 17.9 Gb and 5.71 million reads in four individual experiments. The obtained reads were aligned to the latest mouse reference genome (GRCm38.p6) using the BWA-MEM program, which supports long-read sequencing data. In total, 92.7% of all reads were mapped to the reference genome, and the percentage of Y chromosome reads among mapped reads was 6.59% (Supplementary Table S1). The error rate of mapping reads to the Y chromosome was 12.9% (Supplementary Figure S1). For long-read sequences, the number of reads is not proportional to the total read length, so we compared the fold coverage for each chromosome (Figure 1B). It was confirmed that several specific chromosomes, including the Y chromosome, were enriched. When the sequencing data of four experiments were combined, the fold coverage of the reads mapped to the Y chromosome was 16.8x, the mean length was 4.10 kb, the N50 length was 8.05 kb, and the total length was 1.54 Gb, with a maximum of 113 kb (Figures 1C–H and Supplementary Table S1).

### Genome Assembly

**FIGURE 2:**
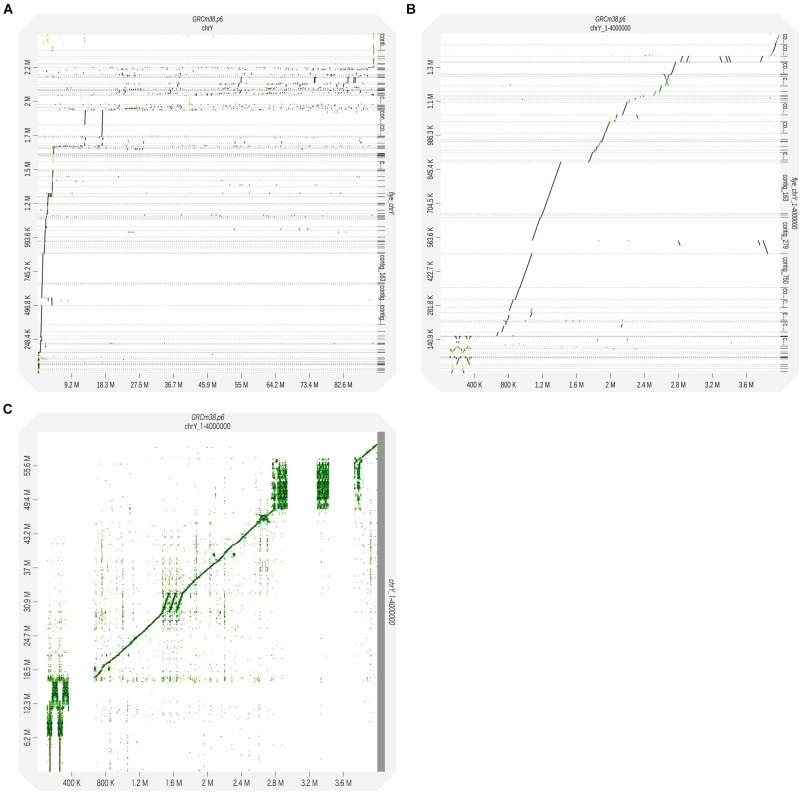
The reads obtained through MinION sequencing were assembled using the Flye assembler. The generated contigs were corrected for error using Racon to obtain the consensus sequences. The polished contigs were used as query sequences and mapped to the reference genome (GRCm38.p6) using BWA-MEM. The alignment results were visualized using D-GENIES. Dot plots were generated to illustrate the pairing between the sequences of the Y chromosome and contigs. **(A)** The polished contigs were compared with the whole sequence of the reference Y chromosome: 1−91,744,698 bp. **(B)** The polished contigs were compared with the reference Y chromosome: 1–4,000,000 bp. **(C)** The sequences of the raw reads with the MinION sequencing were compared with the reference Y chromosome: 1–4,000,000 bp.

Next, we generated a *de novo* assembly to seek the undetermined region. The assembly of overlapping sequences obtained by Nanopore sequencing can reveal unknown sequences of the Y chromosome. *De novo* assembly was performed using the raw long-read sequencing data. We used Flye, an algorithm suitable for assembling long-read sequencing data with a high error rate (Kolmogorov et al., 2019). To identify regions not in the reference, all reads were used, not just the mapped reads. A total of 4,199 contigs were created, and these assembled contigs were polished using Racon, a fast and high-quality consensus module (Vaser et al., 2017), giving 2,594 polished contigs. These contigs were mapped to the reference genome using BWA-MEM. Of them, 216 contigs were thought to be derived from the Y chromosome. The N50 of the reads mapped to the Y chromosome was 42.1 kb, the longest read was 214 kb, and the total length was 2.49 Mb (Supplementary Table S2). In order to compare created contigs with the reference genome, we plotted using D-GENIES (Cabanettes and Klopp, 2018). Although we compared the Y chromosome sequence with contigs mapped to the Y chromosome, the dot plots were fragmented on the long arm of the Y chromosome due to highly repeated sequences (Figure 2A). Therefore, we focused on the short arm from the telomere to the centromere region (1–4,000,000 bp), and it was shown that the assembled contigs were plotted continuously (Figure 2B). Of the deletions seen in Figure 2B, the regions between 1 and 110, 363, and 663 kb, 2.94 and 3.29 Mb, and 3.43 and 3.73 Mb represent the gaps. In contrast, the 1.47–1.71 Mb region is known as a segmental duplication. There were no contigs corresponding to this duplicated region, but the raw reads generated by MinION sequencing were successfully mapped within it (Figure 2C).

### Filling the Gap Regions of the Y Chromosome

**FIGURE 3:**
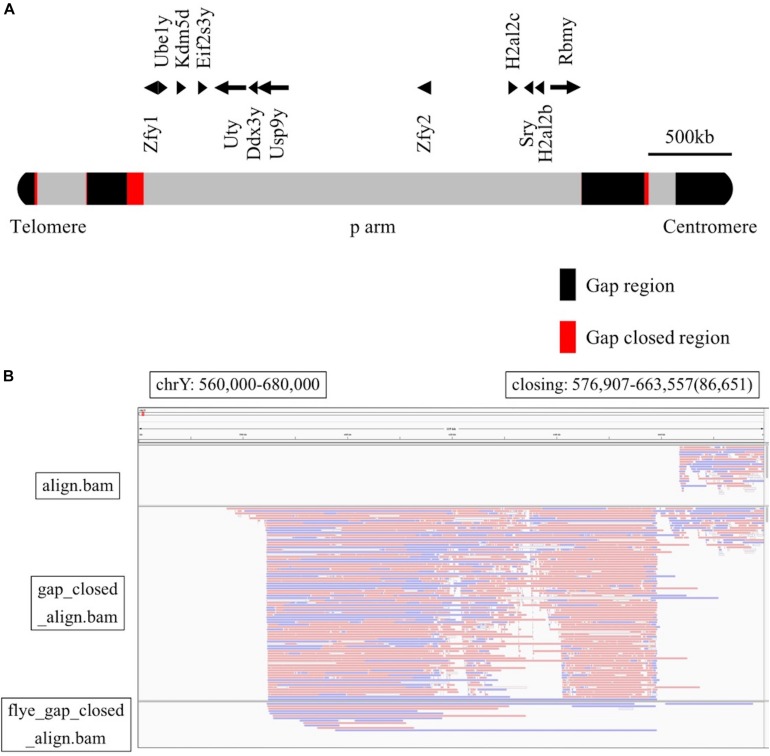
**(A)** The position of Y chromosome genes and gap-closing on the short arm. The gap regions are black, the gap-closed regions are red, and other regions are gray. **(B)** Comparison among the mapping reads to the GRCm38.p6 reference genome (upper row), the mapping reads to the gap-closed genome (middle row), and the mapping of contigs to the gap-closed genome (lower row). This was visualized with the Integrative Genomics Viewer, with the range of the Y chromosome from 560,000 to 680,000 bp.

Finally, to analyze unknown regions of the mouse Y chromosome, we performed sequence extensions at the end of the gaps of the reference genome using long-read sequencing data with LR_Gapcloser (Xu et al., 2019). All read files of the four experiments were used along with the GRCm38.p6 Y chromosome as a reference. Since the long arm of the mouse Y chromosome contains several sequences consequent to chromosome 3 transposition (Soh et al., 2014), we used all reads to identify unknown sequences without any bias. We also performed sequence extensions at the end of the gaps using the reads mapped to just the Y chromosome of GRCm38.p6 with LR_Gapcloser (Supplementary Table S3). The similarity between the two sequences created was high for most regions (Supplementary Table S4). The total Y chromosome gap consisted of 30 regions, including the telomere part, and the total length was 3.62 Mb. Using LR_Gapcloser, we obtained a total of 308 kb gap-closing sequences, which covers 8.5% of the total gap sequences. The newly created sequences were located at 30 sites and had varying lengths, with 86.7 kb being the longest and 241 bp the shortest (Table 1). We compared the newly created Y chromosome sequence with the mouse MSY sequence from Soh et al. (2014), the sequences were almost identical (Supplementary Table S5). Focusing on the short arm of the Y chromosome, newly created sequences were observed at five locations (Figure 3A). Moreover, the new reference genome was created by replacing the Y chromosome of the reference genome with the gap-closed Y chromosome. We mapped the raw reads and the contigs assembled by Flye to the gap-closed genome using BWA-MEM (Supplementary Tables S6–S8) and showed the changed mapping using the Integrative Genomics Viewer (Robinson et al., 2011). It was confirmed that one or more raw reads were mapped in all the gap-closed regions (Figure 3B and Supplementary Figure S2).

**TABLE 1:** Gap regions within the mouse Y chromosome and new regions created by LR_Gapcloser and the number of nucleotides.

Start (bp)	End (bp)	Gap length (bp)	Start (bp)	End (bp)	Filled length (bp)
1	110,000	110,000	96,412	110,000	13,589
363,558	663,557	300,000	363,558	368,103	4,546
2,939,417	3,289,416	350,000	576,907	663,557	86,651
3,429,742	3,729,741	300,000	2,939,417	2,940,255	839
4,469,271	4,529,270	60,000	3,268,142	3,289,416	21,275
11,858,532	11,918,531	60,000	4,469,271	4,477,710	8,440
12,659,191	12,667,190	8,000	4,527,950	4,529,270	1,321
16,539,593	16,599,592	60,000	11,858,532	11,889,503	30,972
18,128,699	18,189,698	61,000	11,913,028	11,918,531	5,504
18,777,490	18,848,489	71,000	16,539,593	16,541,098	1,506
21,706,914	21,756,913	50,000	16,548,870	16,599,592	50,723
22,220,944	22,394,943	174,000	18,128,699	18,131,186	2,488
23,409,944	23,459,943	50,000	18,189,031	18,189,698	668
36,527,797	36,627,796	100,000	18,777,490	18,778,125	636
36,962,067	37,062,066	100,000	18,845,620	18,848,489	2,870
46,063,347	46,163,346	100,000	21,706,914	21,707,989	1,076
49,166,909	49,216,908	50,000	36,962,067	36,972,189	10,123
49,351,015	49,401,014	50,000	37,061,480	37,062,066	587
52,129,007	52,192,006	63,000	46,063,347	46,064,211	865
56,576,231	56,716,230	140,000	46,154,375	46,163,346	8,972
56,891,294	56,941,293	50,000	52,190,686	52,192,006	1,321
57,934,008	57,983,007	49,000	56,576,231	56,582,456	6,226
63,557,161	63,623,160	66,000	56,708,324	56,716,230	7,907
65,001,643	65,031,642	30,000	56,931,900	56,941,293	9,394
66,012,703	66,019,702	7,000	57,981,954	57,983,007	1,054
66,188,542	66,288,541	100,000	63,612,125	63,623,160	11,036
66,452,982	66,502,981	50,000	66,012,703	66,013,383	681
69,457,735	69,538,734	81,000	66,498,576	66,502,981	4,406
87,340,086	87,370,085	30,000	69,526,431	69,538,734	12,304
90,844,699	91,744,698	900,000	87,369,845	87,370,085	241
	Total	3,620,000		Total	308,221

## Discussion

In this study we analyzed the mouse Y chromosome using the Oxford Nanopore MinION sequencer in combination with lasers equipped for chromosome sorting. The use of chromosome sorting for MinION sequencing provides an advantage in time and cost-efficiency. Sequencing technology has improved dramatically, making possible the analysis of large amounts of data at a low cost. The Oxford Nanopore MinION is a third-generation sequencer with low initial cost and long sequence reading without PCR amplification. Theoretically, the repeated and the undetermined regions of the mouse Y chromosome could be identified by long reads obtained by MinION sequencing. However, the error rate of the MinION sequencing would be 5–15% (Rang et al., 2018).

The contigs assembled using Flye did not map well to the Y chromosome, especially on the long arm. It has been reported that the long arm contains about 200 units of a 500kb repeated sequence and that most of the mouse Y chromosome sequences are occupied by segmental duplications (Soh et al., 2014; Morgan and Pardo-Manuel de Villena, 2017). Because of the high sequence similarity (more than 99.9%) among the segmental duplications, which occupy most of the mouse Y chromosome sequences, these sequences were mistakenly recognized as a single sequence. In contrast, the contigs mapped to chromosomes 15 to 18 were obtained in similar numbers, and the total length of these contigs almost completely covered each chromosome (Supplementary Table S2). As a result, it was confirmed that the contigs were mapped to the entire region of chromosomes 15 to 18 (Supplementary Figure S3). To determine the reference sequence of the Y chromosome, including the gap and repeated regions obtained by long reads, it is necessary to develop an appropriate assembly algorithm and improve the accuracy of the long-read sequencer.

Using this method, we obtained the mouse Y chromosome sequences with 16.8× coverage. As the PI used for chromosome sorting binds DNA without base-pair selectivity, chromosomes of similar size could not be completely separated. However, considering that the Y chromosome is haploid and small compared to other chromosomes, the mouse Y chromosome was enriched. Previous research has reported that Chromomycin A3 could be used for sorting with higher resolution than PI and Hoechst33342 staining (Kuderna et al., 2019). However, excitation and detection of Chromomycin A3 require a 457 nm laser and 490 nm LP filter, respectively, and cell sorters that can be equipped with a 457 nm laser are limited. The method presented in this study using common UV and blue 488 nm lasers can be widely applied to enrich for a specific chromosome, including the Y chromosome. A recent report has also suggested that PI and DAPI, as well as Chromomycin A3 and Hoechst33342, can be used for chromosome enrichment (Ng et al., 2019). On the other hand, it is difficult to completely prevent chromosome fragmentation during chromosome isolation by FACS and subsequent DNA purification. Thus, it is important to be very careful during the preparation of chromosomes from M phase-arrested cells and during DNA purification. Additionally, it has been reported that Y chromosomes can be enriched by Y chromosome-specific antisense DNA probes (Alvarez-Cubero et al., 2018). Although our study focuses on the Y chromosome, other chromosomes can be enriched in the same way, and chromosome sorting is thought to be useful for isolating specific chromosomes.

We applied the LR_Gapcloser tool to analyze the undetermined sequences of the reference Y chromosome. Long reads as footholds are indispensable for closing the gap. Therefore, long-read sequencing by Oxford Nanopore MinION is considered to be most suitable for *de novo* genome assembly. However, it appears that a much higher number of reads is necessary to completely fill a reference with a large genome size and a large number of repeated regions. Moreover, it seems to be difficult to obtain single-base resolution, such as SNP resolution, using Nanopore sequencing because of the high error rate.

It has long been thought that elucidation of the precise sequence and structure of autosomes and X chromosomes will reveal genes and genomic regions linked to various diseases. Although there are many concerns that should be addressed regarding the Y chromosome, long-read sequencers, including Oxford Nanopore MinION, may contribute to unraveling the sequence and structure of the Y chromosome. This would shed light on genes and genomic regions on the Y chromosome involved in diseases and abnormalities such as male infertility (Colaco and Modi, 2018). Furthermore, long-read sequencers are powerful instruments for analyzing satellite repeats scattered in the genome and the abnormal repeat expansions seen in neurodegenerative diseases (Ebbert et al., 2018; Cechova et al., 2019). Therefore, long-read sequencers such as the Oxford Nanopore MinION enable the research required to provide new insights into the role of the Y chromosome in health and disease.

## Data Availability Statement

All MinION sequencing data generated in this study have been deposited in the DDBJ Sequence Read Archive (DRA) under the accession number DRA009290.

## Author Contributions

YY planned and performed the experiments, analyzed the data, and drafted the manuscript. TC assisted in performing the experiments, analyzing the data, and preparing the manuscript. HA conceived and supervised the study and critically reviewed the manuscript.

## Conflict of Interest

The authors declare that the research was conducted in the absence of any commercial or financial relationships that could be construed as a potential conflict of interest.

## Supplementary Material

FIGURE S1 Error rate of mapping reads to the Y chromosome using AlignQC. Error rate was calculated by comparison of the raw reads of MinION sequencing and the BAM file mapped using BWA-MEM to the reference genome (GRCm38.p6).

FIGURE S2 Comparison between mapping reads to the GRCm38.p6 reference genome (upper row) and to the gap-closed genome (lower row) for the other 29 regions. This was visualized with the Integrative Genomics Viewer. The red dashed lines were added to denote the existing end-gap sequence of the reference Y chromosome.

FIGURE S3 The polished contigs used as query sequences were mapped to the reference genome (GRCm38.p6) using BWA-MEM. The alignment results were visualized with D-GENIES. Dot plots were generated to illustrate matches between contigs and sequences of the chromosomes 15 to 18. **(A)** Chromosome 15. **(B)** Chromosome 16. **(C)** Chromosome 17. **(D)** Chromosome 18.

TABLE S1 Summary of MinION sequencing for four runs mapped using BWA-MEM. Total reads from individual experiments were mapped to each chromosome. The largest number of reads was mapped to chromosome 17, which is almost the same length as the Y chromosome.

TABLE S2 Summary of Flye assembly mapped to the GRCm38.p6 genome using BWA-MEM. The contigs assembled using Flye were mapped to the reference genome. The number of contigs mapped to the Y chromosome was not very different from that of chromosomes 15 to 18, but the average length and the maximum length were shorter and could not be mapped to the entire region.

TABLE S3 New regions created using the reads mapped to the Y chromosome of GRCm38.p6 by LR_Gapcloser and the number of nucleotides for each. We obtained a total of 269 kb of gap-closing sequences, which covered 7.4% of the total gap sequences. The newly created sequences were located at 27 sites and had varying lengths, with 71.0 kb being the longest and 32 bp the shortest.

TABLE S4 Summary of the similarities between the sequences created using all the reads and the reads mapped to the Y chromosome alone with LR_gapcloser. The sequences at 21 corresponding positions were compared using BLAST (https://blast.ncbi.nlm.nih.gov/Blast.cgi). At most positions, the sequences showed high similarity.

TABLE S5 Summary of the similarities between the newly created sequence and the mouse MSY sequence from Soh et al. The sequences at 10 corresponding regions were compared using BLAST. At most positions, the sequences showed high similarity.

TABLE S6 Summary of the reads that mapped to the newly created genome using all the available reads with LR_Gapcloser. The reads of the MinION sequencing were mapped to the genome, replacing the Y chromosome from the reference genome with the newly created Y chromosome built using LR_Gapcloser.

TABLE S7 Summary of the reads that mapped to the newly created genome using the reads that only mapped to the Y chromosome of GRCm38.p6 with LR_Gapcloser. The new Y chromosome was created by LR-Capcloser using only the reads mapped to just the Y chromosome of GRCm38.p6. The whole reads were also mapped against a second new genome.

TABLE S8 Summary of the reads that mapped to the genome with the mouse MSY sequence from Soh et al. There were no significant differences in the number of reads mapped to the created genome compared to those mapped to the GRCm38.p6 genome.

DATA SHEET S1 Source codes used in this study.
