# Supplementary figures and images for: Analysis of the Mouse Y Chromosome by Single-Molecule Sequencing With Y Chromosome Enrichment
Source: Front Genet. 2020 May 7;11:406. doi: 10.3389/fgene.2020.00406 (PMC7221202; doi:10.3389/fgene.2020.00406)

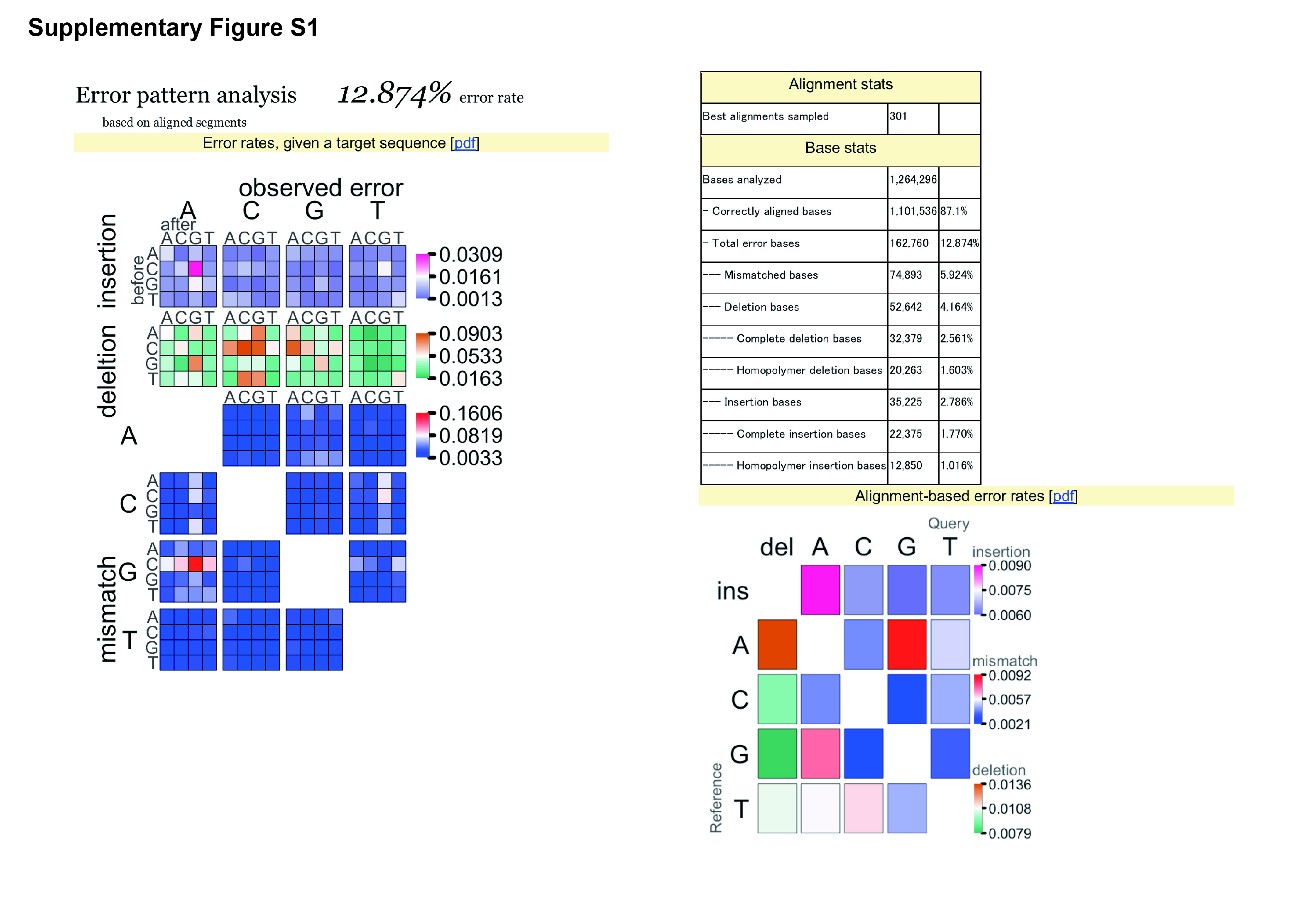

Supplement: FIGURE S1 — Error rate of mapping reads to the Y chromosome using AlignQC. Error rate was calculated by comparison of the raw reads of MinION sequencing and the BAM file mapped using BWA-MEM to the reference genome (GRCm38.p6). [file Image_1.jpg]

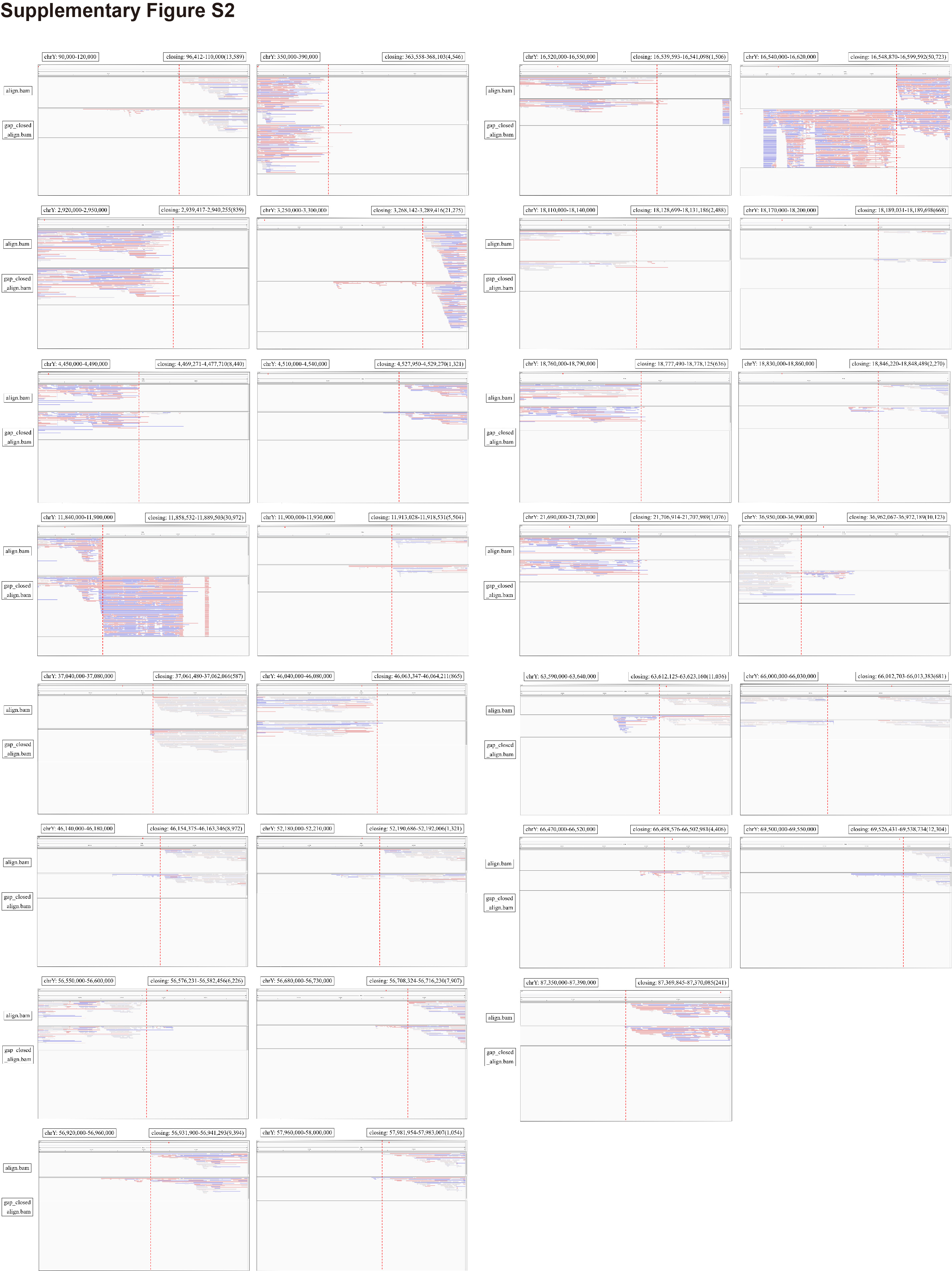

Supplement: FIGURE S2 — Comparison between mapping reads to the GRCm38.p6 reference genome (upper row) and to the gap-closed genome (lower row) for the other 29 regions. This was visualized with the Integrative Genomics Viewer. The red dashed lines were added to denote the existing end-gap sequence of the reference Y chromosome. [file Image_2.jpg]

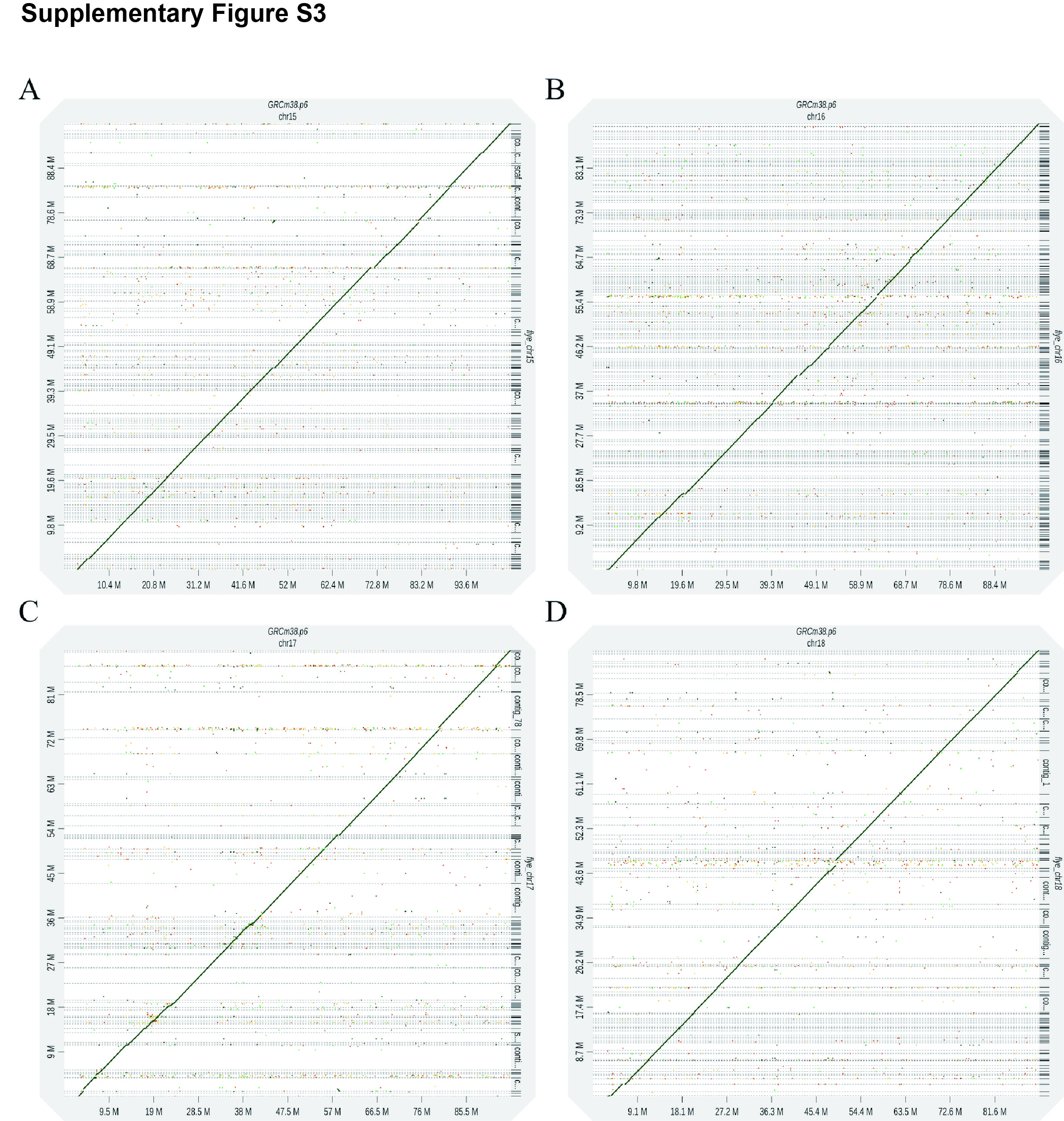

Supplement: FIGURE S3 — The polished contigs used as query sequences were mapped to the reference genome (GRCm38.p6) using BWA-MEM. The alignment results were visualized with D-GENIES. Dot plots were generated to illustrate matches between contigs and sequences of the chromosomes 15 to 18. (A) Chromosome 15. (B) Chromosome 16. (C) Chromosome 17. (D) Chromosome 18. [file Image_3.jpg]
