# Supplementary material for: Analysis of the Mouse Y Chromosome by Single-Molecule Sequencing With Y Chromosome Enrichment
Source: Front Genet. 2020 May 7;11:406. doi: 10.3389/fgene.2020.00406 (PMC7221202; doi:10.3389/fgene.2020.00406)
Supplement: DATA SHEET S1 — Source codes used in this study. [file Data_Sheet_1.docx]

Guppy v3.1.5.

guppy_basecaller --input_path fast5 --save_path basecalled --flowcell FLO-MIN106 --kit SQK-LSK108 --num_callser 4 -x “cuda:0” -r

BWA-MEM v0.7.1

bwa mem -x ont2d GRCm38.p6_genome.fa reads.fastq > align.sam

AlignQC v2.0.5

alignqc analyze chrY.bam -g chrY_GRCm38.p6_genome.fasta --output_folder chrY_alignqc

Flye v2.4.2

flye --nano-raw reads.fastq --genome-size 92m --out-dir flye_out

minimap2 v2.17

minimap2 -x map-ont assembly.fasta reads.fastq > reads_mapped.paf

Racon v1.4.3

racon reads.fastq reads_mapped.paf assembly.fasta > racon.fasta

LR_Gapcloser

bash LR_Gapcloser.sh -i chrY_GRCm38.p6_genome.fasta -l reads.fasta -s n -r 8 -t 8
